# Systematic Assessment of COVID-19 Pandemic in Bangladesh: Effectiveness of Preparedness in the First Wave

**DOI:** 10.3389/fpubh.2021.628931

**Published:** 2021-10-21

**Authors:** Priom Saha, Jahida Gulshan

**Affiliations:** Institute of Statistical Research and Training, University of Dhaka, Dhaka, Bangladesh

**Keywords:** COVID-19, pandemic, Bangladesh, SARS-CoV-2, first wave

## Abstract

**Background:** To develop an effective countermeasure and determine our susceptibilities to the outbreak of COVID-19 is challenging for a densely populated developing country like Bangladesh and a systematic review of the disease on a continuous basis is necessary.

**Methods:** Publicly available and globally acclaimed datasets (4 March 2020–30 September 2020) from IEDCR, Bangladesh, JHU, and ECDC database are used for this study. Visual exploratory data analysis is used and we fitted a polynomial model for the number of deaths. A comparison of Bangladesh scenario over different time points as well as with global perspectives is made.

**Results:** In Bangladesh, the number of active cases had decreased, after reaching a peak, with a constant pattern of death rate at from July to the end of September, 2020. Seventy-one percent of the cases and 77% of the deceased were males. People aged between 21 and 40 years were most vulnerable to the coronavirus and most of the fatalities (51.49%) were in the 60+ population. A strong positive correlation (0.93) between the number of tests and confirmed cases and a constant incidence rate (around 21%) from June 1 to August 31, 2020 was observed. The case fatality ratio was between 1 and 2. The number of cases and the number of deaths in Bangladesh were much lower compared to other countries.

**Conclusions:** This study will help to understand the patterns of spread and transition in Bangladesh, possible measures, effectiveness of the preparedness, implementation gaps, and their consequences to gather vital information and prevent future pandemics.

## Introduction

Severe acute respiratory syndrome coronavirus (SARS-CoV-2) causes coronavirus disease 2019, widely known as COVID-19 (1). COVID-19 is the third emergence of the virus related to severe acute respiratory syndrome (SARS). SARS in 2002–2003 and the Middle East respiratory syndrome (MERS) (2012-present) are the first two inceptions of the coronavirus disease (2). The Coronaviruses were first described in 1966 (3).

Tyrrell and Bynoe first described Coronaviruses as enveloped single-stranded large RNA viruses, those infects humans and a number of animals, and were cultivated from a high proportion of patients with cold (3). Corona is a Latin word meaning crown. With a core shell of spherical virions (entire virus particles), the coronavirus has a surface projection like a solar corona. There are mainly four subfamilies of coronaviruses (alpha, beta, gamma, and delta coronavirus). Alpha and Beta coronavirus originated from mammals (particularly Bats) and Gamma and Delta coronaviruses originated from pigs and birds. From the seven subtypes of coronavirus infecting humans, the beta-coronavirus causes serious fatalities and the gamma-coronavirus causes mild infection. SARS-Cov-2 is a type of beta-coronavirus (4).

After its first spread in Wuhan, the capital city of China's Hubei province on December 1, 2019, the infectious COVID-19 started spreading globally (4). With only one confirmed COVID-19 patient globally on 30 December 2019, the number of patients increased to 219 on 20 January 2020, and on February 24, 2020, the total number of cases increased to 79,565 cases globally. After February 2020, the exponential growth of the infectious virus is still irresistible (4, 5). On January 30, 2020, the World Health Organization (WHO) declared the outbreak a Public Health Emergency of International Concern (PHEIC) and named it a Global pandemic on March 11, 2020 (6, 7). Up to September 30, 2020, more than 214 countries and territories, and 33.5 million confirmed cases of COVID-19 have been reported all over the world (4). About 1 M deaths were caused by the virus during this period and 25.9 million people recovered from COVID-19 (4, 5, 8).

Bangladesh observed the first COVID-19 cases on March 8, 2020, as reported by the Institute of Epidemiology, Disease Control and Research (IEDCR), Bangladesh (9, 10). The country observed the first death due to Covid-19 on March 18, 2020 (9, 10). The deceased was a 70-year-old man who had comorbidities including cardiac problems, high blood pressure, kidney diseases, and diabetes (9). Bangladesh faced a total of 50,000 confirmed cases on June 1, 2020; 100,000 on the 18th of June, 150,000 on the 1st of July, 200,000 on the 17th of July, 300,000 on the 25th of August, and 350,000 confirmed cases on the 20th of September. Up to September 30, Bangladesh tested, a total of 1.95 million samples and 364.9 thousand of them were reported positive cases. A total of 277 thousand people recovered and 5,272 died of COVID-19 during that period (11).

Li et al. analyzed the first 425 cases from Wuhan, Hubei Province, China to ascertain the epidemiological characteristics of the COVID-19 patients (12). The median age of the cases was 59 years, 56% of the confirmed cases were male and the mean incubation period was 5.2 days; the elderly and the patients with other coexisting conditions had higher morbidity (12).

As an infectious disease, primarily the virus spreads between the people who are in close contact and the affected persons have some common symptoms like fever, cough, shortness of breath and loss of sense of smell, dyspnea, headache, sore throat, and rhinorrhea, etc. (13). Studies revealed that the transmission dynamics of COVID-19 is based on two mechanisms such as human to human transmission which is measured with density of population and air pollution to human which is the airborne viral infectivity. People of all ages are at risk, but elderly people and people with pre-existing medical conditions are at greater risk (14, 15). Studies found that COVID-19 related deaths are highly associated with being male, greater age, and medical conditions like diabetes, obesity, cardiovascular diseases, and severe asthma (13, 16).

Several studies reported association between COVID-19 and climatic factors (17–19). Studies showed that, environmental pollutants has significant correlation with COVID-19 patients (20, 21). Population density, temperature and absolute humidity affects the spread of the outbreak (22). A negative association was found between wind speed and covid-19 cases (23). Cities with high air pollution and high atmospheric stability has higher number of COVID patients (24, 25). Also there are apparent differences in terms of COVID-19 response among different countries probably because of their different histories, cultures and political systems and hence there is no straightforward model that could be designated as an Asian or a Western model (26, 27). Countries with low population density as well as efficient and non-corrupted progressive Governments and high health care spending achieved quicker success as compared to the countries not having such characteristics (28, 29).

At the beginning of the outbreak in Bangladesh, the country severely lacked the preparedness to tackle the spread of COVID-19 with both short and long-term implications for health as well as the economy and good governance (30) and that the health care facilities in Bangladesh are inadequate to deal with the pandemic (31). The fragile healthcare system will be in unprecedented pressure with COVID-19 pandemic, in presence of climate hazards such as floods, heat waves, etc. and disease outbreaks like dengue, cholera, and diarrhea (32). On the other hand, during an ongoing pandemic, the livelihood opportunities may reduce to a significant extent resulting in partial or complete loss of income with a significant change in the financial status or consumption behavior (33, 34). Under such circumstances, increased health care practices such as mobile sanitization, temporary quarantine sites, health care facilities, and empathic collaborations between government and locals were suggested (35) instead of a continuation of complete lockdown or shutdowns to reduce the problem.

Strong implementation of the lockdown resulted in success for several countries. New Zealand, being one of the successful eliminators of the COVID-19 pandemic, started implementing their influenza plan in early February, 2020 (36). The state of Kerala in India used their prior experience of handling the Nipah virus through extensive testing, contract testing, and community mobilization resulted in controlled spread of COVID-19 (37). Taiwan and South Korea also suppressed the COVID-19 disease successfully by quickly responding to the disease and with clear and consistent decisions to combat the threat (38).

Every outbreak and health emergencies provides a window of opportunity to gain knowledge and to develop an effective countermeasure and determine our susceptibilities to those measures. Literature shows that most of the countries that succeeded in controlling the pandemic has used their previous experience in handling the infectious disease. So a systematic review of the COVID-19 pandemic in Bangladesh on a continuous basis is always necessary and a proper analysis of the effectiveness of the preparedness, transparency of the situation and knowledge sharing can ease out the way to make a safer future.

Unfortunately, although the first wave of Covid-19 pandemic had severe impact of health of people, a large number of countries were neither capable enough to make an efficient national planning nor timely application of the best practices for management of crisis (39). We believe that the lessons learned from the first phase of COVID-19 from the perspective of Bangladesh will be insightful while Bangladesh is experiencing another wave of Covid-19 as well as future pandemics.

What this study adds to the current literature is a systematic analysis of the overall patterns in terms of number of cases, number of deaths, and impacts of Coronavirus disease in Bangladesh, a developing country. The death patterns of Bangladesh and other countries as well as the trends over time were analyzed to compare the COVID 19 situation of Bangladesh with those countries. This study focused light on the underlying causes that resulted in a continuous outbreak. The preparedness, effectiveness of the preparedness and possible steps amid further waves are also suggested in this study.

## Methods

### Sample and Data

The data for the daily tests, cases, recoveries, deaths, and age-specific death rates are collected from the daily press release of the Directorate General of Health Services, Ministry of Health & Family Welfare of Bangladesh (10). The demographic distribution of the cases is collected from The International Data Rescue (I-DARE) portal (40). The daily number of reported new cases of COVID-19 by country worldwide is collected from the European Centre for Disease Prevention and Control (ECDC) and Johns Hopkins University (JHU) database (41). The data are extracted from 4 March 2020 to 30 September 2020.

### Measures of Variables

The data consists of the number of confirmed COVID-19 cases, deaths, recoveries, daily tests, total test, and demographic characteristics (Age, Sex) of the cases and deaths.

### Data Analysis Procedure

**Figure 1:**
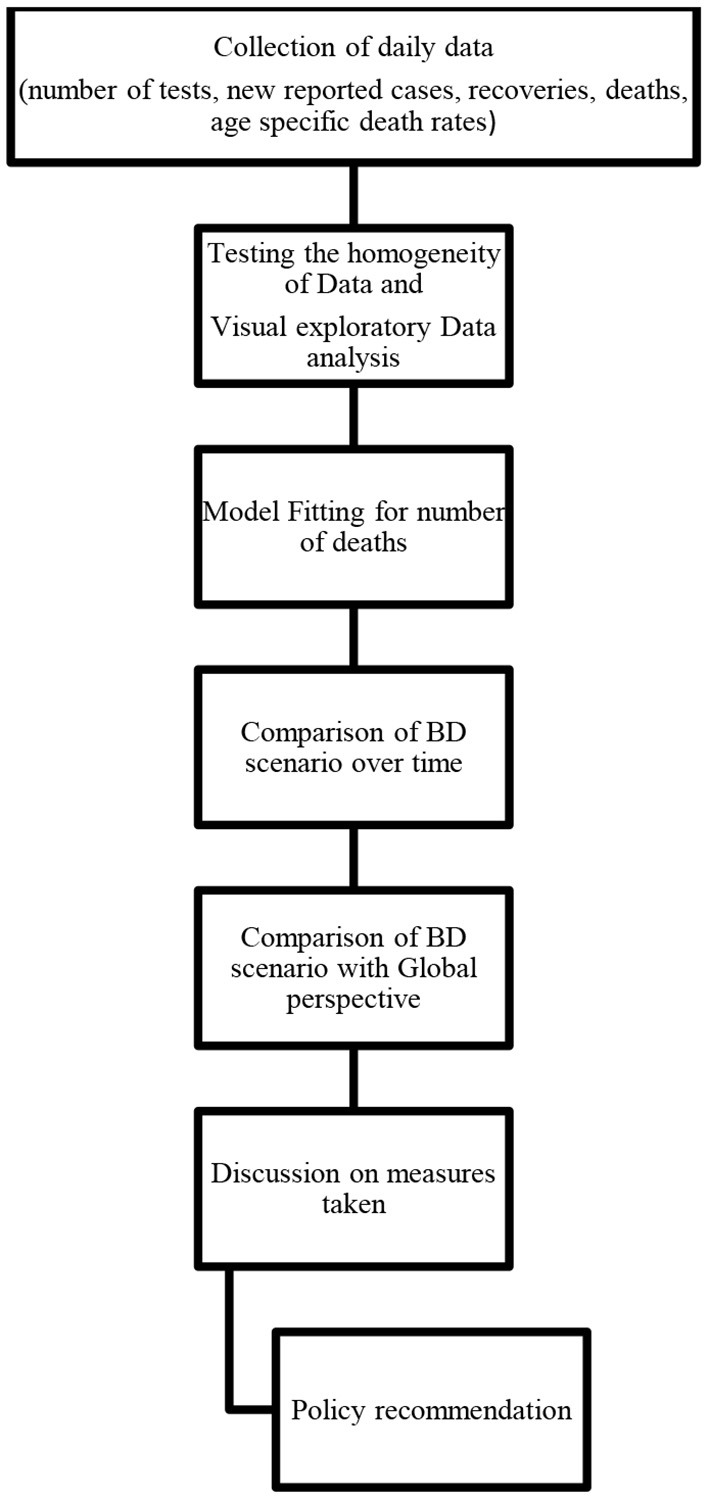
Systematic flowchart.

Visual exploratory data analysis (V-EDA) is used to analyze the characteristics of the COVID-19 pandemic situation in Bangladesh. We have compared the situation in Bangladesh with other countries using several EDA tools such as scatter plots, histograms, bar plots, and geographical representation is shown by plotting the administrative maps using R package “mapReassy” (42). A systematic flow chart given below (Figure 1) summarizes the methodology of this study.

To test the inhomogeneity's in the data, in absolute homogeneity methods, we performed Normal Homogeneity Test (SNHT) (43), Buishand range test (44), Pettitt's test for single change-point detection (45), and Von Neumann ratio test (46) for number of confirmed cases and number of deaths. The alternative hypothesis, for the first three tests equals existence of a stepwise shift, whereas Von Neumann test checks the randomness in the data. The results shows the data is heterogeneous and not randomly distributed. The test results are added in the Table A3 (Supplementary Material).

### Model

In absence of homogeneity, we proposed a curvilinear trend (6th order polynomial) for deaths in Bangladesh. The order of the model is selected based on goodness of fit measures. We used Akaike's Information Criteria (AIC) and Bayesian Information Criteria (BIC) to select the model.

The regression equation is given by:


(1) $$\begin{array}{l} \text{Y}={\text{β}}_{0}+{\text{β}}_{1}^{*}x+{\text{β}}_{2}^{*}x^{2}+{\text{β}}_{3}^{*}x^{3}+{\text{β}}_{4}^{*}x^{4}+{\text{β}}_{5}^{*}x^{5}+{\text{β}}_{6}^{*}x^{6}\ldots . \end{array}$$

- Y = No of deaths; x = No of days.
- The incidence rate, Case fatality are calculated as follows:
- Incidence Rate = cases ^*^100,000 / (161.4^*^1,000,000)
- Case-Fatality Ratio (CFR) (%) = Number recorded deaths^*^100 / Number of cases.

## Results and Discussion

The trends in cases, recoveries, mortality, and active cases are studied thoroughly to capture the actual picture in Bangladesh. In addition to the situational reports from Bangladesh Government and the World health organization (WHO), this study highlighted the prime aspects of the first wave of COVID-19 situation in Bangladesh.

**Figure 2:**
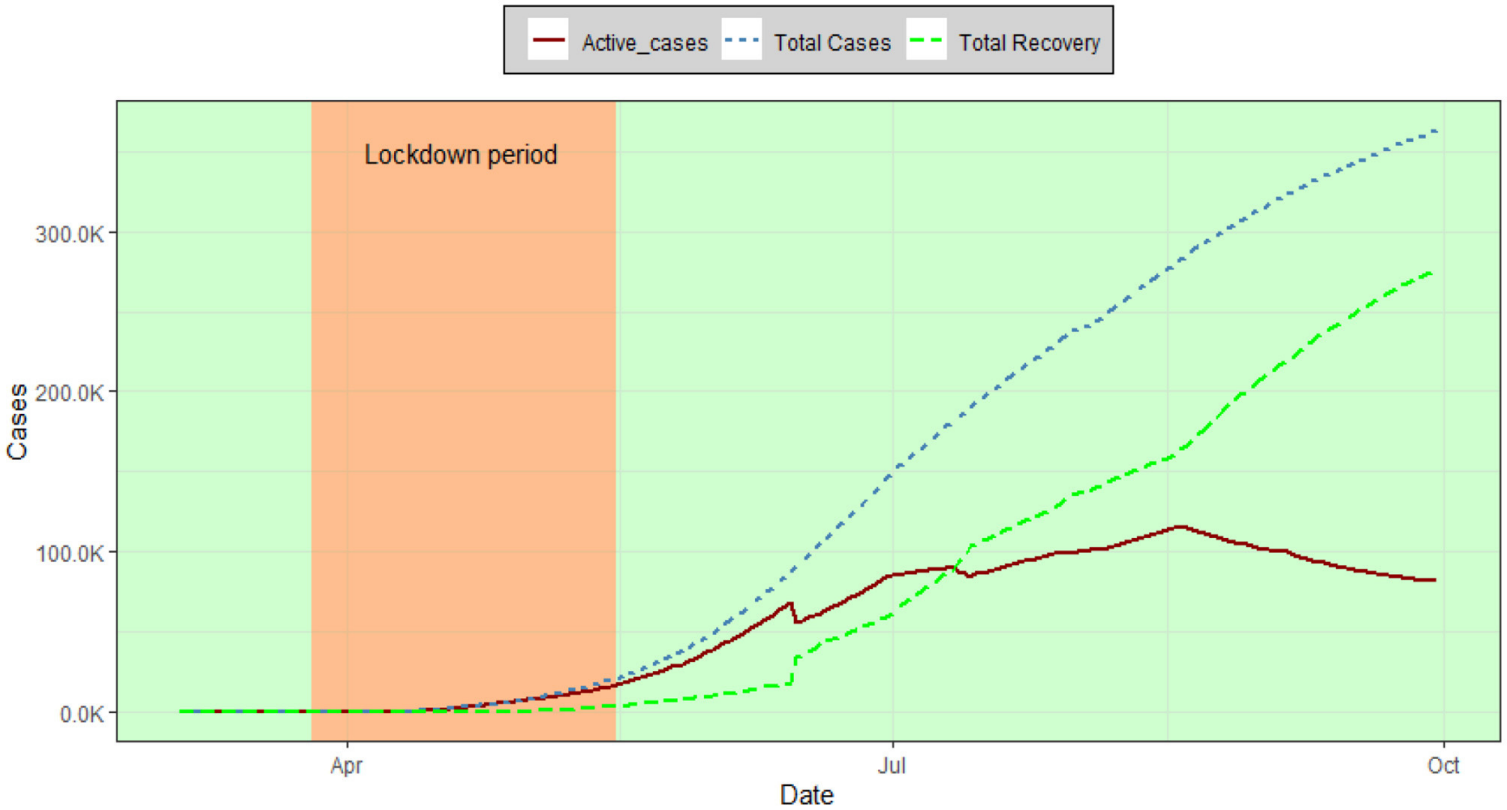
Total cases, recovery, and active cases over time in Bangladesh.

**Figure 3:**
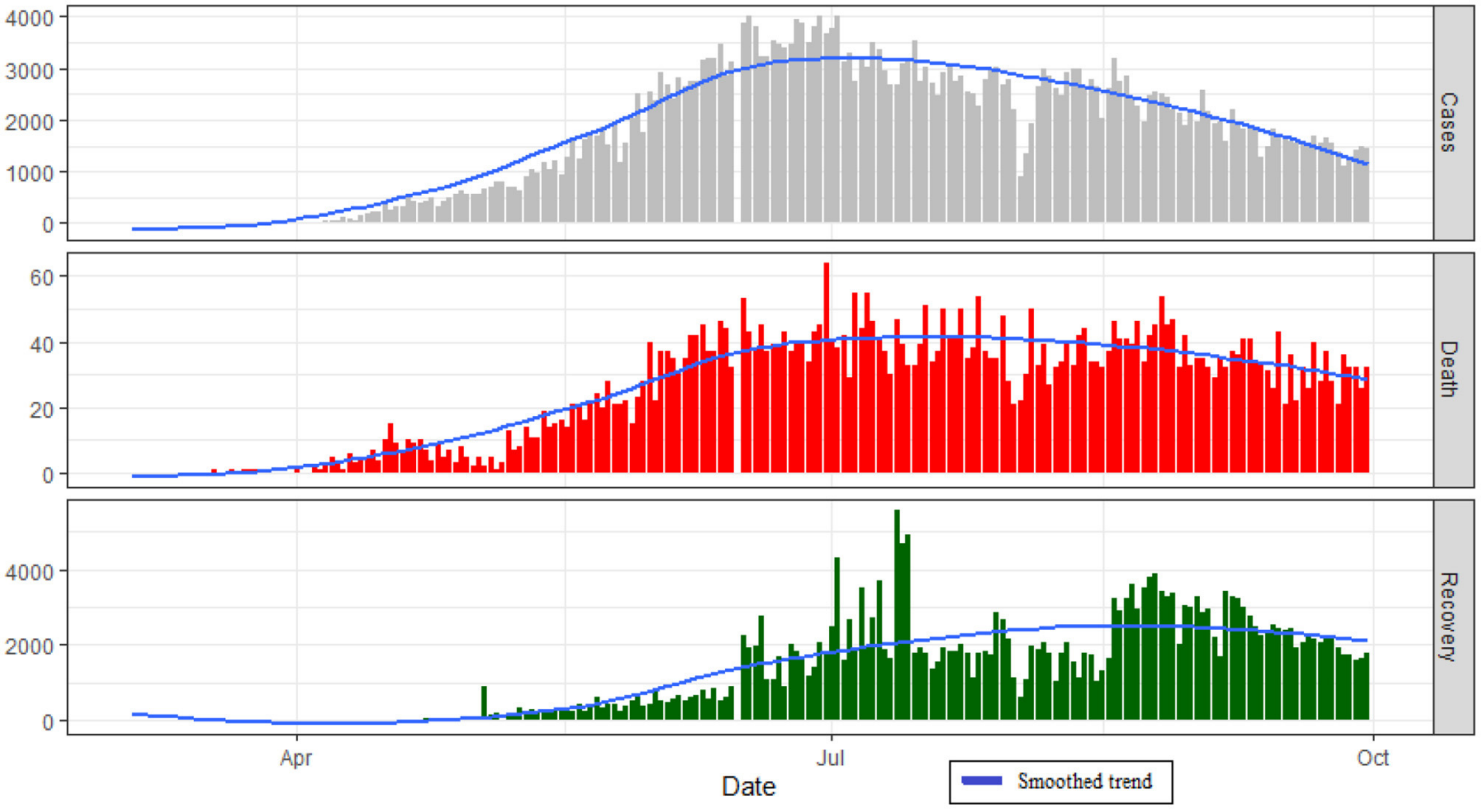
Daily change in cases, recovery, and death in Bangladesh.

Figure 2 reflects the overall trend of COVID-19 patients in Bangladesh. Up to September 30, the total recovered patients in Bangladesh have increased resulting in a decreasing pattern in the number of active cases. The number of confirmed cases followed an increasing pattern from March to June and decreased afterward (Figure 3). The number of Recovered people was increasing over time from the last week of June and thereafter.

**Figure 4:**
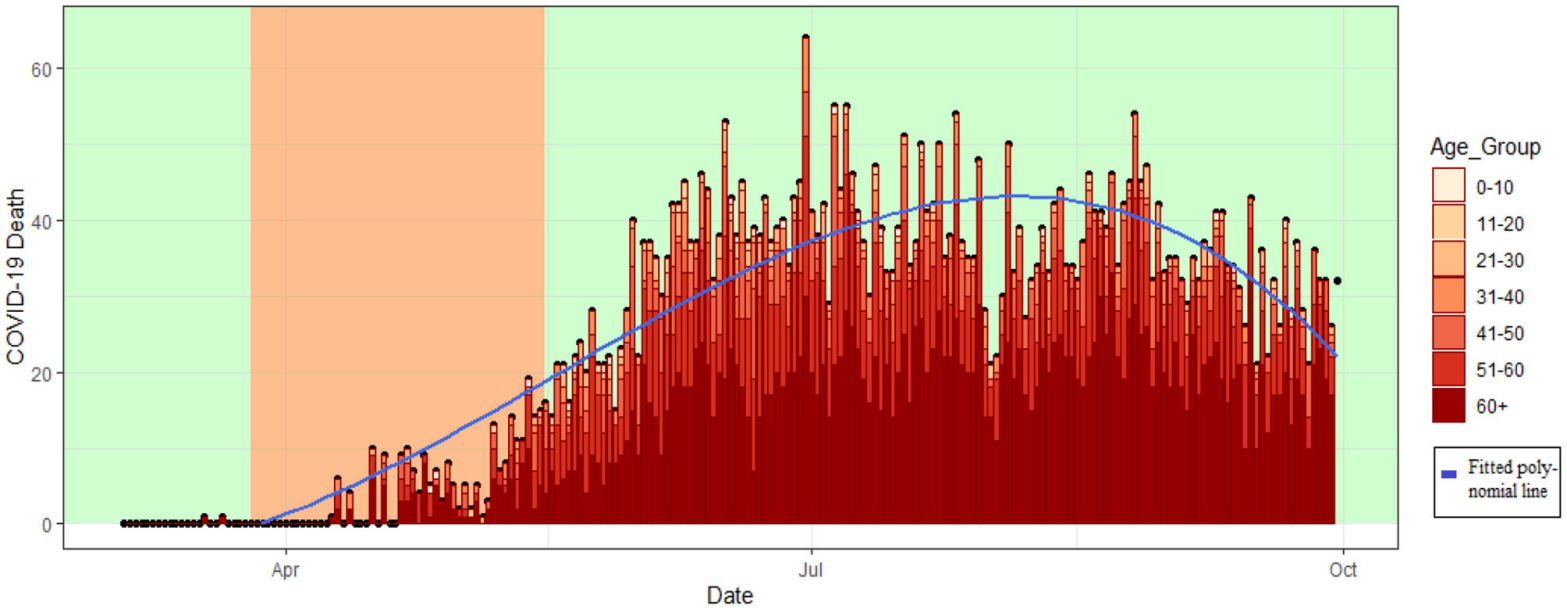
Death trends over time in Bangladesh.

The number of COVID 19 deaths in Bangladesh has increased over time from March to mid of July (Figure 4). The death rate has a peak in the period from the last week of June to the second week of July with a constant pattern thereafter.

The deaths were limited in the lockdown period (26th March 2020 to 16th May 2020) and the death toll raises rapidly after that. From June to September the deaths among the younger people increased alongside the elderly deaths (Figure 4). The death trend in Figure 4 are fitted using 6th order polynomial regression (Equation 1) and a detailed table is attached in the Table A3 (Supplementary Material).

**Figure 5:**
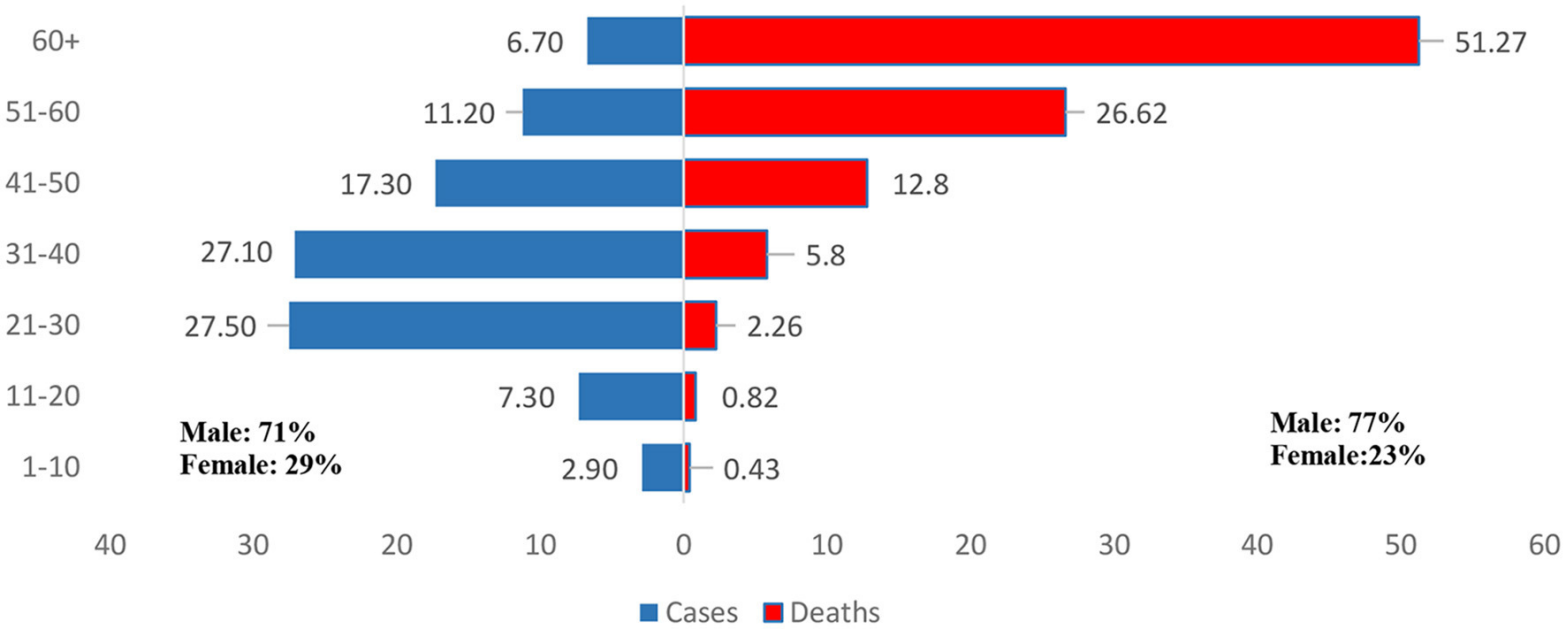
Age distribution of cases and deaths in Bangladesh.

The demographic characteristics of the COVID-19 patients indicate (Figure 5) that young people aged from 21 to 40 were the most affected by novel coronavirus disease and 71% of the affected were males are rest were women (29%). In the case of deaths, the elderly people (60+) were the most vulnerable and 77% of the deaths were among the male (Female 23%) (Figure 5).

In Bangladesh, the number of male cases outnumbered the number of female cases (Male: 71%, Female: 29%). Also the number of male deaths outnumbered the same of female deaths (Male: 77%, Female: 23%). Similar pattern was observed among the South Asian countries (for example India, Pakistan, Nepal, and Afghanistan) [a detailed table is attached in the Table A1 (Supplementary Material)]. Although the world data suggests both males and females are equally likely to corona-virus-disease (47). However, the gender role in mortality is also observed in SARS patients (14). In the pandemic period including the lockdown period the males more prone to roaming outside as they are the bread earners mostly, resulting exposure. Study also suggests higher tobacco consumption rate and comorbidity in males are some of the reasons (48).

**Figure 6:**
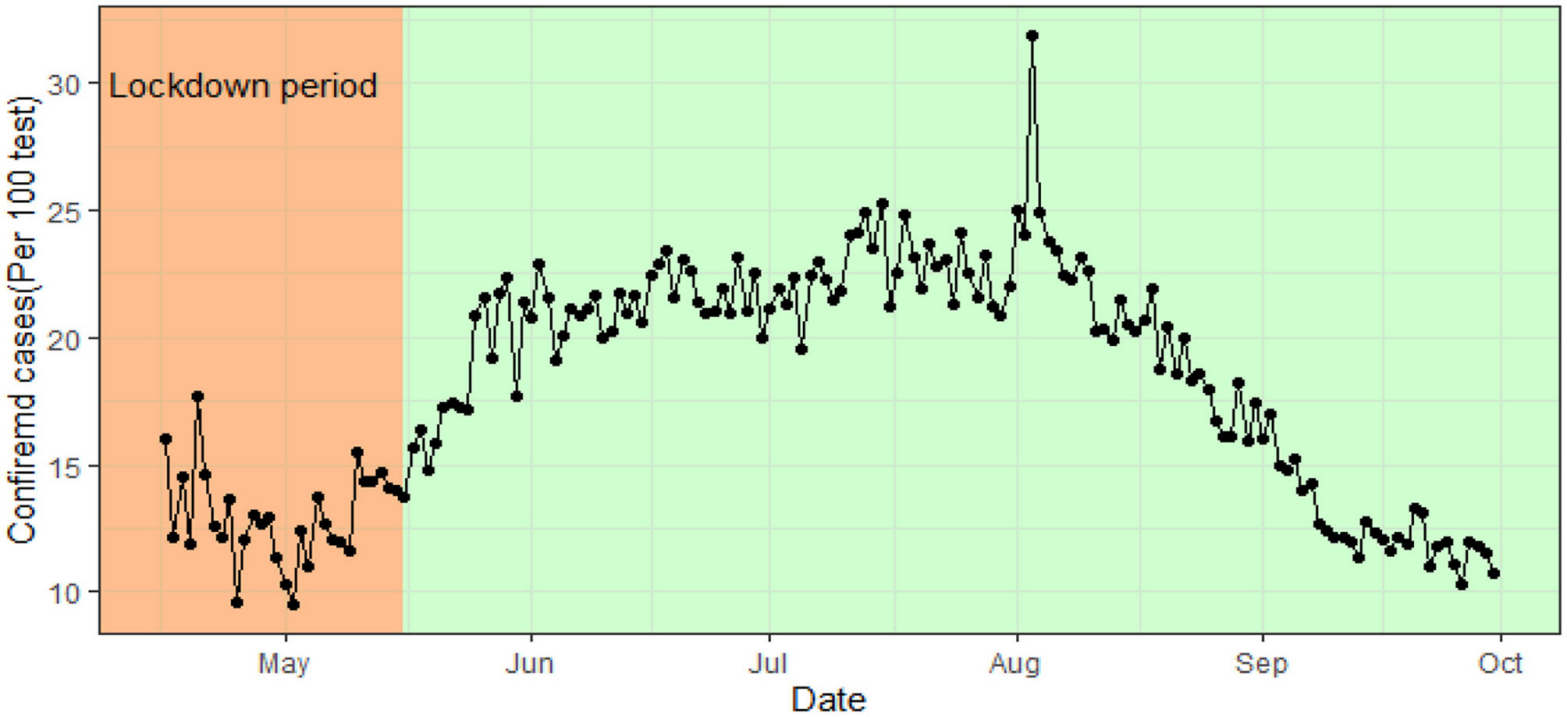
Cases per test (100) rate in Bangladesh over time.

**Figure 7:**
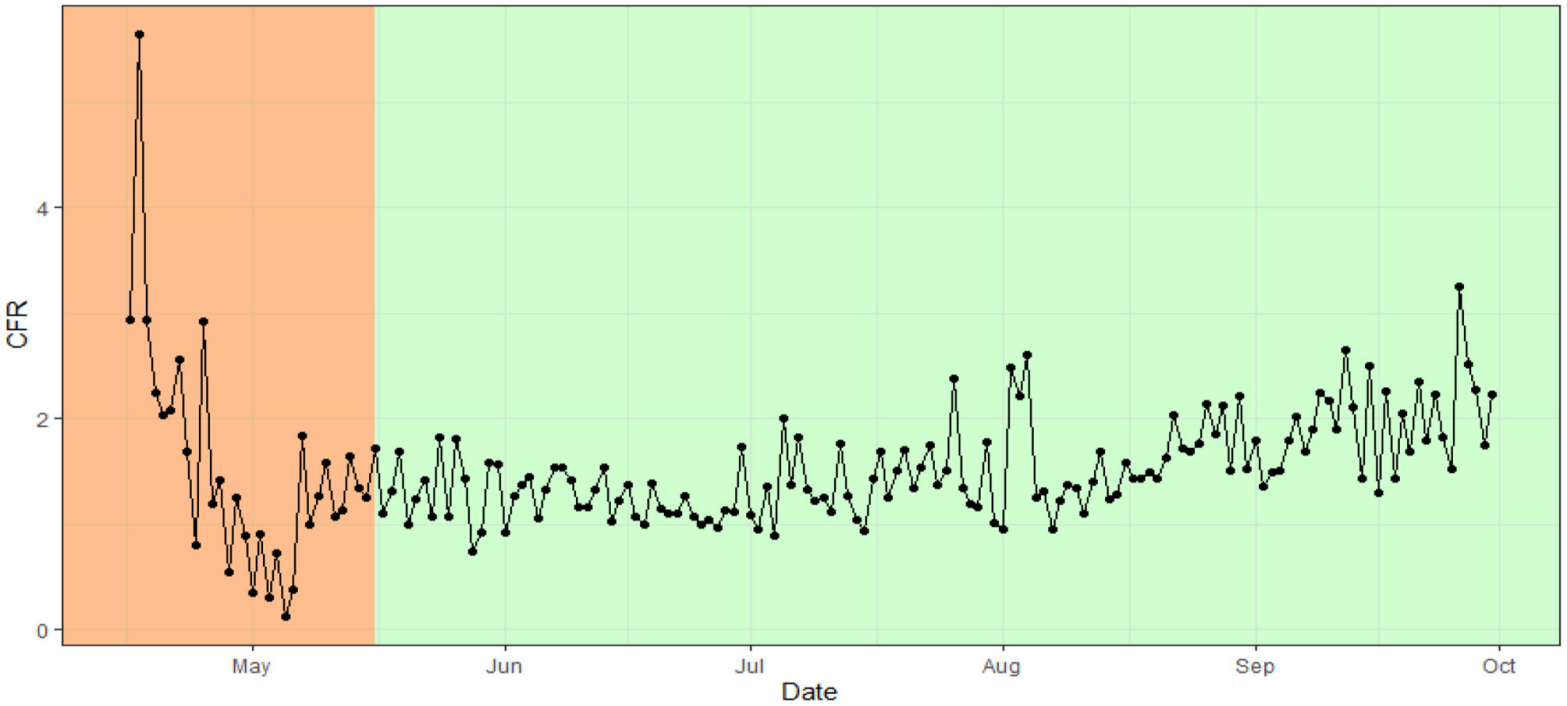
Case fatality ratio in Bangladesh over time.

The COVID-19 confirmed cases in Bangladesh were below 15% in the lockdown period (from 26th March to 16th May). From June 1 2020 to August 31 the rate remains constant at around 21%. That is, around 21 persons were tested COVID-19 positive per 100 test.This constant rate over time indicates a dubious incidence rate (Figure 6). Whereas, after the initial decrease of the case fatality ratio (CFR) in the lockdown period the CFR remains constant in between 1 and 2% with an average of 1.53. That is, out of 1,000 confirmed cases around 15 people die due to COVID-19 (Figure 7).

**Figure 8:**
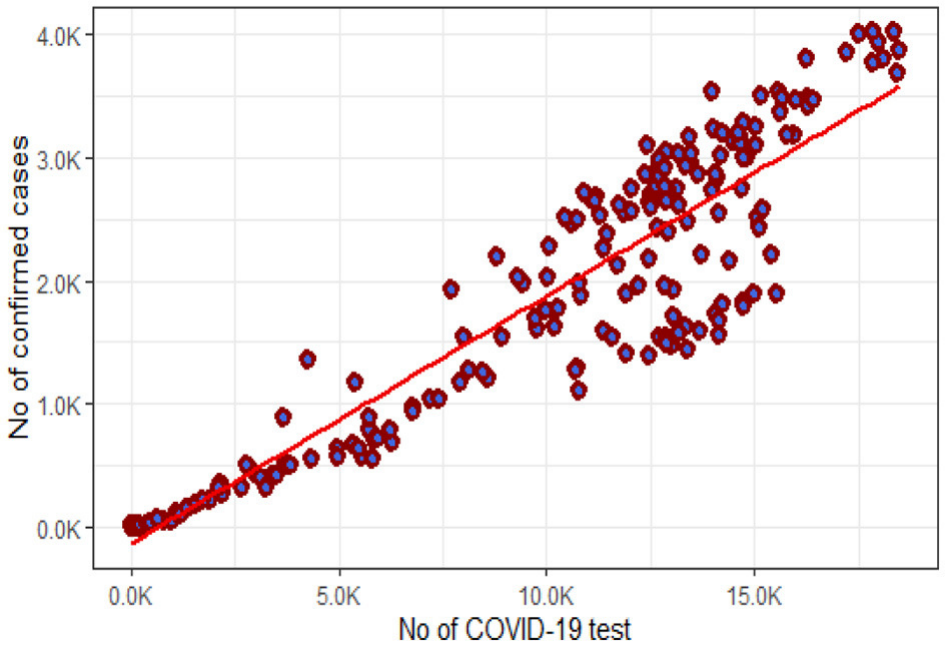
Association between number of COVID-19 tests and confirmed cases in Bangladesh.

The statistics on the number of positive COVID cases and consequently recoveries and deaths are depending on the number of tests. In Bangladesh, the incidence rate was following a constant rate (Figure 5) indicating under-reported number of positive cases. High correlation (0.93) between test and positive cases indicates if the test could be conducted in higher numbers, than the actual incidence rate could have been captured (Figure 8).

**Figure 9:**
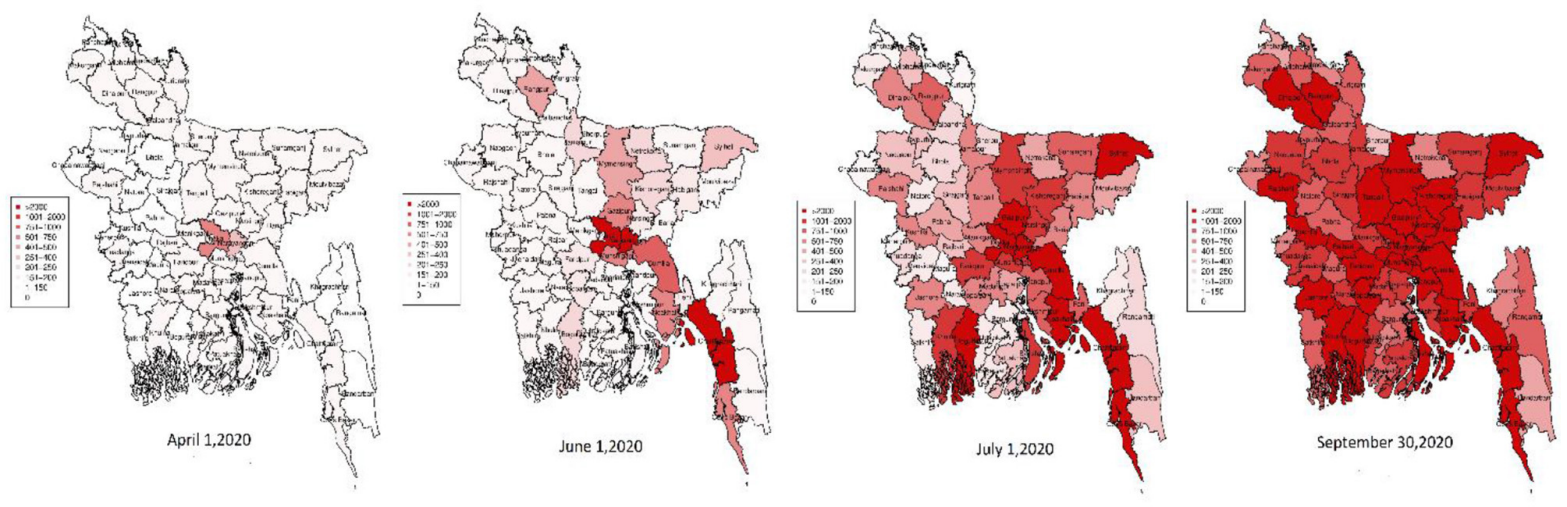
Spread of COVID-19 positive cases in Bangladesh.

In Bangladesh, the infectious virus spread from some particular cities (mostly Dhaka) to the whole country. While Bangladesh observed the first COVID-19 confirmed cases on March 8, 2020, on 16th April, the virus affected 44 districts out of 64 districts in Bangladesh. Dhaka and Narayanganj had the most COVID-19 positive cases (Dhaka: 608, Narayanganj: 255). Narayanganj and Dhaka worked as an epicenter for further spread in this time. On the 1st of June, the virus outspreaded mostly Cumilla, Noakhali, Chattagram, Cox's Bazar, Mymensingh, Jamalpur, Rangpur, and Sylhet. The worst-hit districts are the eastern, north-eastern, and south-eastern districts alongside Dhaka district. On the 1st of June, the spread intensified. The number of total confirmed cases increased rapidly thereafter. Upto 28th September, most of the districts have more than 2,000 confirmed COVID-19 cases (Figure 9).

Among the COVID-19 infected countries, Bangladesh was the 117th country in comparison to other countries considering cases per million population till September 30, 2020 [detailed figure is in Figure A1A (Supplementary Material)]. In terms of total cases, the USA observed the most COVID-19 positive cases and Bangladesh was the 16th country in comparison to other countries till September 30, 2020 (Figure A1B in the Supplementary Material). Whereas, in terms of total death per million population, Bangladesh was 111th compared to other countries (Figure A2A in the Supplementary Material). In comparison to the total number of deaths, the United States of America (USA) has the most deaths (2.1 million) and Bangladesh was 29th in total deaths compared to other countries till September 30, 2020 (Figure A2B in the Supplementary Material).

**Figure 10:**
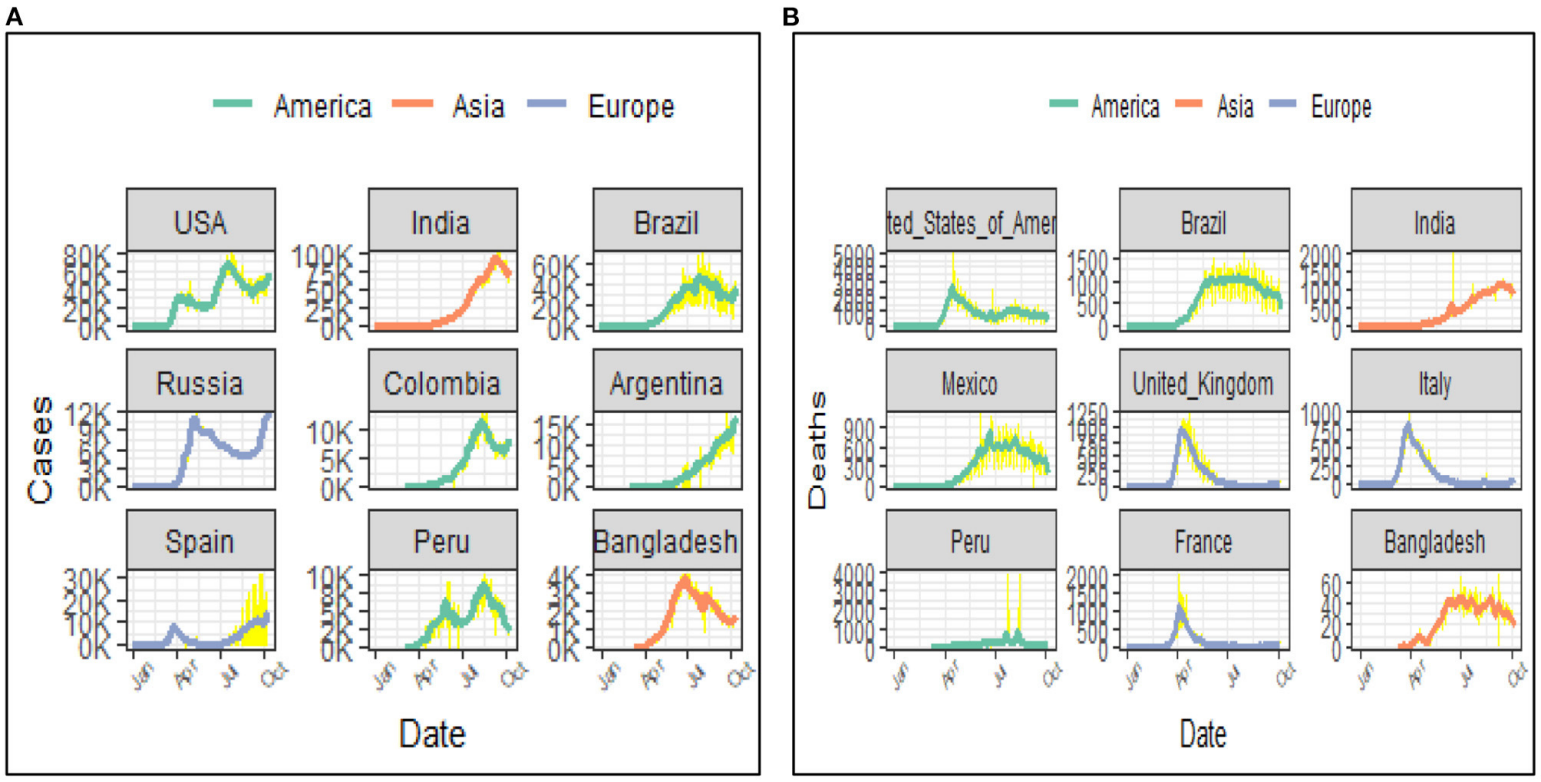
**(A)** Case pattern over time (7 days average) (top 8 countries and Bangladesh). **(B)** Death pattern over time (7 days average) (top 8 countries and Bangladesh).

From the observed case patterns (Figure 10A), in the USA, Russia, and Peru the number of cases started increasing again after decreasing for a period indicating a second wave. Whereas, in Bangladesh India, Brazil, and Colombia the cases were decreasing; indicating high risk of a second wave. The graph (Figure 10B) shows the death pattern in the USA, Brazil, the UK, Italy, and France already reached a peak point and the death rates are decreasing over time. But in India, Mexico, and Bangladesh the death rates were still in the peak position.

### Covid-19 and Measures Taken

For a developing country like Bangladesh, COVID-19 is a challenging catastrophe. Bangladesh observed its peak positions in the number of cases and deaths. The government took numerous measures to fight the COVID-19 pandemic in Bangladesh such as screening, rescuing, and lockdown, restriction on local and international air travels, and switch to online educational activities for students instead of on campus activities.

Fifty-three days after the first identification of COVID-19 in Wuhan, the Dhaka Airport authority started screening the passengers who arrived from China (49). Up to 30th September, Bangladesh has screened a total of 9,87,848 passengers. Among them, 5,61,108 are screened in the International Airports, 3,78,449 in the land ports, 41,262 in the seaports, and 7,029 passengers in the cantonment railway station (10).

The government rescued 312 Bangladeshi citizens from Wuhan on the 1st of February and quarantined them for 2 weeks in the Ashkona Hajj camp for 2 weeks. None of them tested positive (50, 51). On 15th March, 417 Bangladeshi returns from Italy. Two hundred seventy-five of them were kept under government supervision and 142 were sent to home quarantine (52).

Bangladesh declared the first National Preparedness and response plan for COVID-19 on 18th March (53). The first lockdown was declared on 19th March at Shibchar, Madaripur (54). On the 9th of April government imposed a complete “No entry No exist” lockdown in the Cox's Bazar District, where most of the Rohingya refugee camps are located (10, 55).

On the 23rd of March, the government ordered the closure of public and private offices from March 26 to April 4 and extended it to April 14th. On 24th March Armed forces are deployed to ensure social distancing and quarantine (10, 55). On April 10, the first general holidays are announced on April 15–23 and with several further extensions to 31st of May. On 28th May government declared the nationwide shutdown would gradually be lifted conditionally from May 31 to 15th June. All government/semi-government/Autonomous offices will be kept open to a limited extent to maintain the 13 point directive declared by DGHS (10). The transportation is also resumed on a limited scale from May 31 (10).

On 24th March a 10-day ban on all passenger travel by Air, Water, and Rail was imposed (10). Biman Bangladesh suspended all its domestic and international flights on 27th March (56). All domestic flights resumed from the 1st of June (1). Biman Bangladesh started its international flights from June 21, 2020 (52).

All the educational institutions were declared closed on 16th March. The shutdown extended several times till the 31st of October. The Government also postponed the Higher Secondary Certificate (HSC) examination on 22nd March and on 7th October the HSC and equivalent exams were declared canceled by the Educational Minister (57, 58).

The government announced a stimulus package of 8.5b USD, almost 2.5% of GDP on 5th April, 2020, amid COVID-19 impact on the economy (10). A new stimuli package of 589b USD for the agricultural sector was declared on 12th April, 2020 (10). On 20th April, 2020 Bangladesh Bank announced 30b taka lending for low-income groups (10, 55, 59).

Despite government measures, the situation in Bangladesh is deteriorating. Though some studies state that lockdown has a significant impact on reducing the spread (60), some other showed that in general, the countries with a weak health care sector apply lockdown for longer duration but longer period of lockdown does not reduce fatality rate significantly (61) or that delaying reopening does not reduce the magnitude of the second peak of cases, but only delays it (62, 63). Studies on Bangladesh also showed that, in Bangladesh, the lockdown measures were not much effective with no sign of flattening the curve (31). Furthermore, since a longer period of lockdown has a negative impact on economic growth, it is difficult for the people of a developing country like Bangladesh to continue with lockdown for long periods (61). During ongoing pandemic livelihood opportunities has decreased and a lot of people has experienced significant or complete loss of income (34). With a significant changes in consumption pattern and financial situation (33), uncontrolled spread of the COVID-19 pandemic could leave a bulk vulnerable groups in socioeconomic crisis (64). In addition, the ongoing pandemic has a severe physiological impact across Bangladesh especially among the women and younger people (65), whereas, the older adults are more fearful of COVID-19 (66). The terminated lockdown and relaxation in other measures (such as transport restriction, mass gathering restriction, and restriction on people in the mosque at prayer, restriction in super malls, and theaters) to combat this have endangered the situation.

The mass people in Bangladesh are reluctant to the testing procedure. Since the rate of complications due to COVID-19 was low (Serious: 10–15%, Critical: 5% cases), people with moderate symptoms preferred to stay at home and avoided the tests (67). Most of them took telemedicine services from home. According to IEDCR/DGHS, up to September 30, 2020 the total number of phone calls in various hotlines (16,263, 333, and 10,655) was 21.2 million. And among them, 417, 598 receives COVID treatment (10, 40). The costly RT-PCR (3,500 tk at hospitals, 4,500 tk for samples collected from home) was one of the major reasons for the reluctance to test. Besides, the poor management in the testing booths, poor hygiene, and lack of social distancing are the key factors for the reluctance to the testing procedure. There is a firm belief that the negative people could get transmitted in the booths and they prefer not to test.

A study conducted by the IEDCR found around 45% of the Dhaka dwellers are exposed to COVID-19 and carrying antibody (40, 68, 69). Whereas, 9% (nearly 20 Lacks) could be COVID-19 positive with 78% having asymptotic patients (70). Besides 2 out of 3 slum dwellers in Dhaka and Chattagram have had COVID-19 (71). This results indicates the possibility that a small portion of the actual affected patients are reported.

However, studies showed there concern about probable under reporting bias in the COVID-19 data of Bangladesh due to resource constraints (72). Self-reported syndromic data also suggests an earlier spread of the outbreak in Bangladesh (73). Besides, study based on mortality rates found massive under reporting of confirmed cases in many countries (74). Due to limited testing capacities, confirmed cases data are not exact (74). Lack of testing kits and test facility (Lab) is one of the prior reasons for the low number of tests in the initial stage. Up to 30th May, there was only 50 lab facility for COVID testing which is 106 till now (as of 30th September). Besides, the testing facility was mainly Dhaka concentrated initially. The first COVID testing started in IEDCR Dhaka and the government gradually increased the testing facility centering Dhaka. Among the 108 Labs, 57% of labs (60) are in Dhaka as a consequence the testing in other areas is under-reported. According to the IEDCR/DGHS, 50.11% of deaths are reported in Dhaka. Lack of awareness among the mass people and social stigma are the major reasons for low testing in Bangladesh.

In world's perspective, the incidence rate in Bangladesh compared to other countries was apparently controlled during the first wave. Bangladesh had crossed the peak period and the cases were decreasing. While after crossing first peak position, the cases in the USA, Russia, and Peru crossed/reached their second peak. Compared to them the case rates decreased in Bangladesh which was a success. The death rate in Bangladesh was also low compared to many other countries in the world during the period. The death pattern in the USA, UK, France, and Italy has crossed the peak and was moving down during June and afterwards. On the other side, Brazil, Mexico, and Bangladesh was crossing their peak time. And the death rate pattern was clustering at the peak for a large period without decreasing.

The volume of evidence during COVID 19 and at which it evolves had been a big challenge for policy makers all over the world (75). A government can break the chains of transmission and outbreaks using varying restriction policies including quarantine, social distancing, business closures or full lockdown, or a combination of any or all of these measures (38). However, keeping a balance between health and economic objectives always threw challenges to the policy makers.

Going forward, a country must examine the impact of COVID-19 in her own ability in order to continue making significant progress toward Sustainable Development Goals and solutions need to be created together with Policy makers and Public (76). And should plan to build resilient communities (77) and resilient healthcare system (76, 78).

Studies showed that, rather than a longer period of lockdown, greater healthcare expenditures (as % of GDP) can be more helpful to reduce COVID-19 fatality rates and hence an efficient strategy for future pandemics is to increase healthcare investments (61). Mathematical and computational modeling efforts have had an enormous impact on public health policy for the prevention and control of COVID-19 in the US and abroad (79, 80). However, with poor facilities of contact tracing, reluctance of people for mass testing for economic or social reasons, and insufficient investment in health care sector, it is difficult for a developing country like Bangladesh to suggest public health policies based on Mathematical or computations models due to probable underreported data.

Furthermore, studies showed concern regarding public perceptions regarding the COVID-19 pandemic because it might affect the policy makers and because of uncertainty. In addition, the “infodemic” make it even harder for the policy makers to convince people to “follow” the evidence (75). Examples of misleading advice on COVID-19 that had been rapidly and widely spread online creating threats to specific public health measures (including wearing mask or social distancing) were also true for Bangladesh (75). General people are often reluctant and ignore broad public health measures undermining COVID-19 responses.

Environmental factors should also be carefully considered in policy making for Covid-19. COVID-19 is generating substantial amounts of hazardous waste worldwide and Bangladesh is a part of it. In Bangladesh, there is an alarming unhealthy practice of collecting used mask and PPE by the waste collectors who resale it in the local market illegally as reported by the frontline newspapers and TV channels. A poor medical waste management system might increase the risk of spread of Covid-19. On the other hand, a wastewater surveillance for COVID-19 pandemic for inclusion of wastewater based epidemiology in policy making is also very important (81).

Safe mass vaccination is the long term solution for the current pandemic (82). With limited resources of health system of Bangladesh, any vaccine providing a protection, at least against severe COVID-19 cases would reduce the burden on the scanty hospital and intensive care unit facilities in the country (83, 84). Also, the mass participation to vaccination can be ensured through advertising about herd immunity (85). Fortunately Bangladesh Government has given priority to this issue and is moving forward with its vaccination strategy. With an aim of vaccinating 80% of the total adult population, the vaccination drive was inaugurated on 27th January, 2021 (86). The Bangladesh government published a priority list for the first round of vaccine recipients, including frontline workers and older people aged 40 years and above. A compulsory app-based registration system was developed for registration of vaccination against COVID-19. The vaccines, primarily, were distributed through tertiary healthcare centers in the capital city of Dhaka. Another proportion were dispersed through district hospitals and Upazila health complexes (1st referral center at primary healthcare level) (87).

In conclusion, using the lessons learned from the first wave of the crisis of COVID-19 pandemic in Bangladesh and in other countries of the world, this study suggests that, for a developing country like Bangladesh, a long period of lockdown cannot be a suitable measure to control the spread of the pandemic as it might give birth to economic crisis. Rather, social awareness regarding the spread of the disease and its probable impact, usefulness of using mask, as well as a strong healthcare sector might be helpful to fight against COVID-19.

### Limitations

Our study has a few limitations. The publicly available dataset could have some misreported or under reported data. In Bangladesh data, on 15th June there is a sharp increase in the total number of recoveries due to the change in the definition of recovery by IEDCR (a patient can be declared recovered if the fever goes down without paracetamol or similar drugs and if there is a significant improvement in breathing or coughing problems within 3 days) (88). According to DGHS, over 15,000 patients recovered from COVID-19 up to June 15, 2020. The patients who recovered from COVID-19 included both symptomatic and asymptomatic patients which could not be specified due to lack of data. The deaths and recoveries occurred not only in the hospital but also at home. Lack of massive testing and lack of data on such cases, we could not shed light on that part.

## Conclusions

To combat an infectious disease, it is not only important to know about the virus biology but also the nature of the spread, probable measures to prevent the disease, the effectiveness of measures, and the loopholes in those measures. In Bangladesh, the COVID-19 confirmed cases showed an exponential increase after the lockdown was relaxed.

The Government of Bangladesh had taken numerous measures to tackle the pandemic situation and consequently the spread had somewhat been controlled in Bangladesh in the first phase. However, after a long lockdown of 51 days, the “not minimized” incidence rates are probable indicators of implementation gaps of measures taken by the Government. No proper screening of the cities with international airports (Dhaka, Sylhet, and Chattagram), delay in starting screening, improper quarantine of the rescued people including the confirmed cases, lack of proper testing facilities, poor contact tracing policies were the major loopholes. In addition to that, social stigma and non-cooperation and reluctance of general people due to lack of proper knowledge regarding the spread of such an infectious disease deteriorated the situation to some extent. It is difficult to track the amount of medical waste managed properly in the current health care system of the country. However, a monitoring might improve the situation and further studies are required in this field.

The government could not succeed completely to enact the importance of the suggested guidelines to prevent the disease among the mass people. As a result, there was poor control over the implementation process to maintain the guidelines. In view of the systematic assessment of the disease in Bangladesh, we suggest that to prevent further spread, strict maintenance of the guidelines [that is, (i) Wearing mask (ii) Sanitization, (iii) Social distancing], proper lockdown policy optimized with respect to economic issues, proper screening in the ports, proper quarantine, proper medical waste management, mass testing, and finally, mass vaccination. However, mass awareness is the mandatory first step to implement any of these measures successfully. We strongly believe that these insights will assist Bangladesh as well as the other densely populated countries fighting against future pandemics.

## Data Availability Statement

Publicly available datasets were analyzed in this study. This data can be found here: https://drive.google.com/drive/folders/1nQ4SM_D97lyKehWnRmTX7y8yy9U7Qujf?usp=sharing.

## Ethics Statement

Ethical review and approval was not required for the study on human participants in accordance with the local legislation and institutional requirements. Written informed consent from the participants' legal guardian/next of kin was not required to participate in this study in accordance with the national legislation and the institutional requirements.

## Author Contributions

JG has conceptualized the study and critically reviewed and revised the manuscript. PS collected and compiled data and drafted the manuscript. JG and PS read and approved the final manuscript. Both authors contributed to the article and approved the submitted version.

## Conflict of Interest

The authors declare that the research was conducted in the absence of any commercial or financial relationships that could be construed as a potential conflict of interest.

## Publisher's Note

All claims expressed in this article are solely those of the authors and do not necessarily represent those of their affiliated organizations, or those of the publisher, the editors and the reviewers. Any product that may be evaluated in this article, or claim that may be made by its manufacturer, is not guaranteed or endorsed by the publisher.
