# Supplementary material for: Systematic Assessment of COVID-19 Pandemic in Bangladesh: Effectiveness of Preparedness in the First Wave
Source: Front Public Health. 2021 Oct 21;9:628931. doi: 10.3389/fpubh.2021.628931 (PMC8567082; doi:10.3389/fpubh.2021.628931)
Supplement: Supplementary file 1 [file Data_Sheet_1.docx]

# Appendix:

**Table-A1:** Distribution of cases and deaths in South Asian countries.

| Country | Cases | | Deaths | |
| --- | --- | --- | --- | --- |
|  | Male | Female | Male | Female |
| Bangladesh | 71% | 29% | 77% | 23% |
| India | 64.56% | - 35.44% | 64% | - 36% |
| Pakistan | 73.98% | - 26.02% | 74.15% | - 25.85% |
| Nepal | 72.93% | - 27.07% | 69.39% | - 30.61% |
| Afghanistan | 70.13% | - 29.87% | 74.74% | - 25.26% |
| Source: The COVID-19 Sex-Disaggregated Data Tracker(49) | | | | |

Table-A2: Lockdown periods in South Asian countries and others.

| Country | Lockdown Started | Lockdown Ended | Days |
| --- | --- | --- | --- |
| Bangladesh | 26^th^ March,2020 | 16^th^ May,2020 | 51 |
| India | 25^th^ March,2020 | 7^th^ June,2020 | 74 |
| Pakistan | 24^th^ March,2020 | 09^th^ May,2020 | 46 |
| Nepal | 24^th^ May,2020 | 21th July,2020 | 120 |
| Afghanistan (Kabul) | 28^th^ March,2020 | 20^th^ May,2020 | 53 |
| New Zealand | 26^th^ March,2020 | 14^th^ May,2020 | 46 |

Table-A3: Heterogeneity test results

| Test Name | **Confirmed case** | | **Deaths** | |
| --- | --- | --- | --- | --- |
|  | Test statistic | Decision | Test statistic | Decision |
| Standard Normal Homogeneity Test (SNHT) | 154.14*** | Reject | 167.19*** | Reject |
| Buishand range test | 6.22*** | Reject | 6.365*** | Reject |
| Pettitt's test for single change-point detection | 10231*** | Reject | 10552*** | Reject |
| Von Neumann ratio test | 0.07072*** | Reject | 0.32168*** | Reject |

*** p-value < 0.001

**Table-A3: Anova Table**

| Term | Estimate | Std. Error | Statistic(t value) | p-value |
| --- | --- | --- | --- | --- |
| Intercept | -3.929e+00 | 1.147e+00 | -3.426 | 0.00063 *** |
| β_0_ | 1.032e | 1.481e-01 | 6.967 | 4.88e-12 *** |
| β_1_ | -6.34E-02 | 6.03E-03 | -10.503 | <2.00E-16*** |
| β_2_ | 1.47E-03 | 1.06E-04 | 13.788 | <2.00E-16*** |
| β_3_ | -1.39E-05 | 9.11E-07 | -15.241 | <2.00E-16*** |
| β_4_ | 5.82E-08 | 3.73E-09 | 15.619 | <2.00E-16*** |
| β_5_ | -9.05E-11 | 5.84E-12 | -15.487 | <2.00E-16*** |

‘***’ <0.001

Adjusted R^2^ = 0.8841

**Trends:**

| 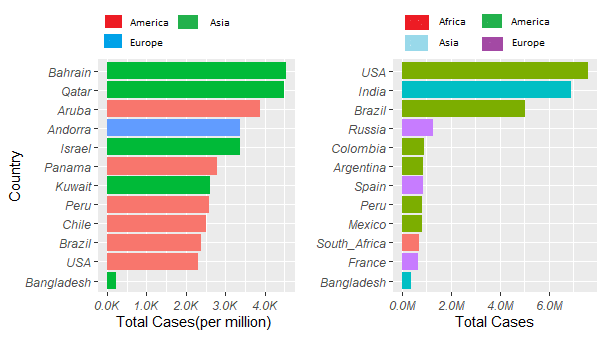 | |
| --- | --- |
| **Figure A1(A):** Top 11 countries and Bangladesh (117^th^) by total cases per million of population. | **Figure A1(B):** Top 11 countries and Bangladesh (16^th^) by total cases. |

| 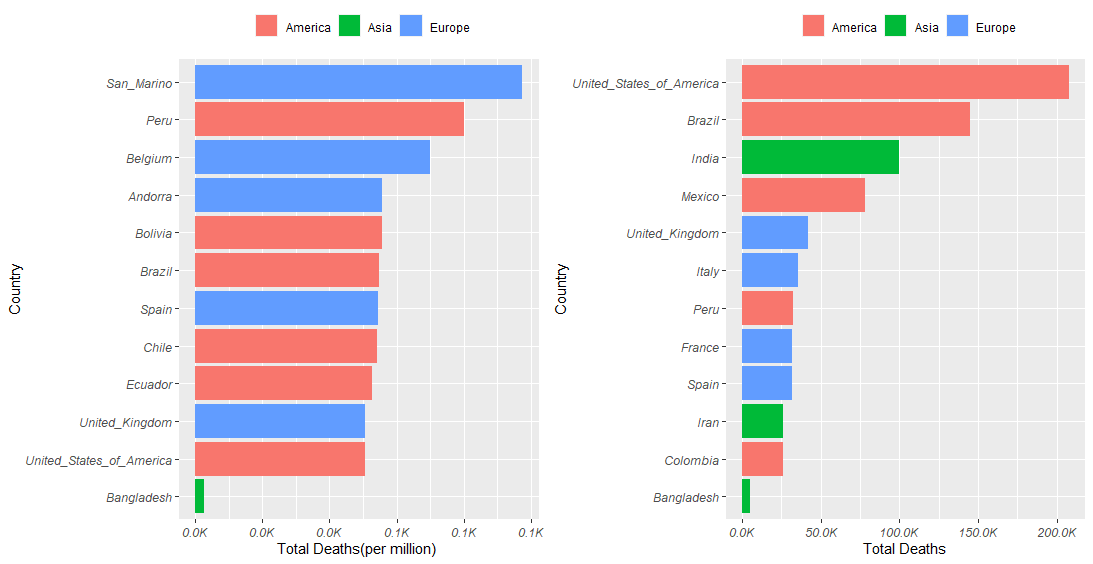 | |
| --- | --- |
| **Figure A2(A):** Top 11 countries and Bangladesh (111^th^) by total deaths per million. | **Figure A2(B):** Top 11 countries and Bangladesh(29^th^) by total death |

| 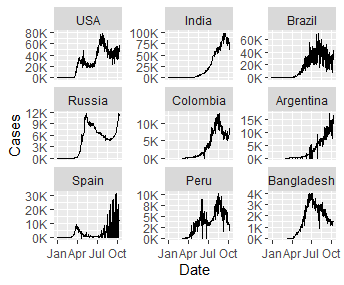  Figure A3(A): Incidence pattern over time (Top 8 countries and Bangladesh) | **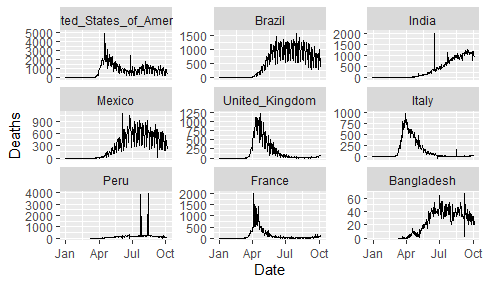**  Figure A3(B): Death pattern over time (Top 8 countries and Bangladesh) |
| --- | --- |
